# *In situ* microscopic observation of chitin and fungal cells with chitinous cell walls in hydrothermal conditions

**DOI:** 10.1038/srep11907

**Published:** 2015-07-07

**Authors:** Shigeru Deguchi, Kaoru Tsujii, Koki Horikoshi

**Affiliations:** 1 Japan Agency for Marine-Earth Science and Technology (JAMSTEC), 2-15 Natsushima-cho, Yokosuka 237-0061, Japan; 2 Graduate School of Engineering, Chuo University, Bunkyo-ku, Tokyo 112-8551, Japan

## Abstract

Recent findings of intact chitin in fossil records suggest surprisingly high recalcitrance of this biopolymer during hydrothermal treatments. We also know in the experience of everyday life that mushroom, cells of which have chitinous cell walls, do not fall apart however long they are simmered. We used *in situ* optical microscopy to examine chitin and fungal cells with chitinous cell walls during hydrothermal treatments, and obtained direct evidence that they remained undegraded at temperatures well over 200 °C. The results show very hot and compressed water is needed to make mushrooms mushy.

Chitin is a linear and nonionic polysaccharide made of *N*-acetylglucosamine units that are connected via β-1-4 glycosidic linkages^1^. It is the most abundant organic polymer in the marine environment^2^ and its annual production amounts to 10^6^–10^7^ tons^3^. Chitin is used as structural components by a broad range of living organisms, including cell walls of fungi, exoskeletons of insects, arthropods, and sponges, and beaks of cephalopods^4^. In addition to living organisms, chitin determines decay and preservation of soft-tissue in fossil deposits^5^,^6^. Intact chitin was found preserved in 25-million-year-old fossil insects^7^ and 34-million-year-old fossil cuttlefish^8^. Recent studies have found preserved chitin even in 200-million-year-old gastropod egg capsules^9^ and 505-million-year-old fossil marine sponge from the Burgess Shale^4^. Given harsh physicochemical conditions to which the Burgess Shale was subjected during diagenesis (temperatures up to 250–300 °C under pressure)^10^, the finding is remarkable and demonstrate the recalcitrance of chitin under extreme physicochemical conditions.

Thermal gravimetrical analysis (TGA) showed thermal degradation of solid chitin of various origins occurred only above 300 °C^11^. What is also relevant in considering formation of fossil records is the recalcitrance of chitin in the presence of water^12^,^13^. Stability of chitin in hydrothermal conditions is also relevant for practical applications^14^. A wide variety of chitin-based inorganic-organic composites, including SiO_2_^15^, ZrO^16^, ZrO_2_^17^, and Fe_2_O_3_^18^, were successfully obtained in hydrothermal reactions. Such unique composites attracted interest in the development of bone substitutes for tissue engineering, waste water treatment, and drug delivery systems^14^. During hydrothermal synthesis, chitin remained undegraded and served as a template on which inorganic nanoparticles deposited. Presumably, the same is true for fossilization of any types of chitin-containing soft-bodied organisms, but basic chemical knowledge on the recalcitrance of chitin in water under extreme conditions has been extremely sparse.

In the case of cellulose, which is a linear and nonionic polysaccharide made of glucose units that are connected via β-1-4 glycosidic linkages and is the primary component of plant cell walls^19^, its resistance to degradation in hydrothermal conditions was successfully studied by using an optical microscope equipped with a high-temperature and high-pressure sample chamber^20^,^21^,^22^. Cellulose remained essentially unchanged at temperatures well above 250 °C when it was heated in water at a constant pressure of 25 MPa, and was eventually hydrolysed at around 300 °C. The stability was ascribed to robust crystalline structure of cellulose^20^, in which extensive hydrogen bonding networks were formed between the cellulose chains, thereby effectively prevented hydrolytic attacks of water molecules at high temperatures.

Structural similarity between chitin and cellulose suggests that chitin likely shows similar stability in water at high temperatures and high pressures to that of cellulose. In this paper, we report *in situ* microscopic observations of chitin and fungal cells with chitinous cell walls in water at high temperatures and high pressures up to the supercritical state of water (*T*_c_ = 374 °C, *P*_c_ = 22.1 MPa)^23^.

## Results

### Observation of chitin in supercritical water

**Figure 1:**
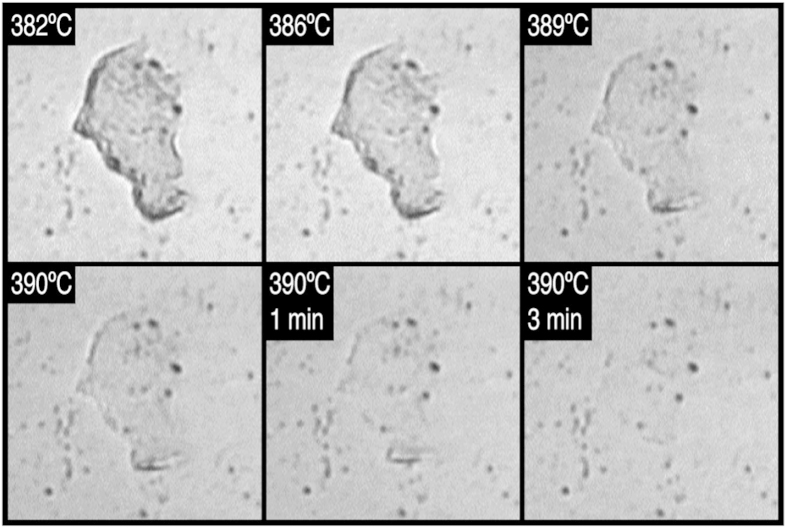
*In situ* optical microscopic images showing dissolution of a flake of chitin in supercritical water. Images were taken under a constant pressure of 25 MPa. Each image is 170 μm × 170 μm. A video clip showing the dissolution process is available in Movie S1.

We first examined the behaviour of chitin in water up to the supercritical state of water (Fig. 1). Flakes of chitin from crab shell (*Chionoecetes japonicus*) were dispersed in water, and introduced to the sample chamber on the microscope. Specimen was then pressurized to 25 MPa and heated while maintaining the pressure. The observation was made while heating the specimen from room temperature up to 390 °C (Video showing entire process is available in Movie S1). We found that chitin was significantly more resistant to hydrothermal degradation than cellulose, and did not observe any noticeable change up to ~380 °C. Above ~380 °C, the flake of chitin gradually became thinner and disappeared completely at 390 °C. The thin flake rolled-up before complete dissolution (Movie S1), suggesting that chitin lost crystallinity and became plastic, just as crystalline cellulose did when it lost crystallinity^20^. Rolling of the chitin flake may also suggest structural heterogeneity inside the flake before dissolution^24^. Same result was obtained in observation of another chitin flake.

Cell walls of fungi, such as yeast and mushrooms, are 80–90% polysaccharide, mostly chitin, and proteins, lipids, and inorganic ions constitute the rest^25^. Cell walls play a decisive role for living cells to withstand physical stresses such as osmotic stress^25^. We know by experience that mushrooms do not fall apart however long they are simmered, showing that their chitinous cell walls remain undegraded for long when it is treated in water at 100 °C. The robustness of chitin in hydrothermal conditions implies that morphology of fungal cells may be preserved in water even at much higher temperatures.

### Morphological change of yeast cells in hydrothermal conditions

To verify this, we examined morphology of cells of an yeast, *Cryptococcus liquefaciens*, in water at high temperatures and at a constant pressure of 25 MPa (Video showing entire process is available in Movie S2). The temperature increased linearly between 50 °C and 230 °C at a rate of 40 °C/min during the observation (Figure S1).

In our experimental set-up, there is a strong convective flow in the sample chamber during heating^21^. The convective flow is so strong that even large objects, several dozens micrometres in size, move around during heating. Accordingly, imaging of small objects such as *C. liquefaciens* cells (approximately 5 μm in diameter) by this instrument is usually difficult^26^. To alleviate the issue, a dispersion of *C. liquefaciens* cells was introduced into the chamber and set at room temperature overnight, during which the cells adhered firmly to the surface of the optical window made of diamond. The cells thus treated were not driven away by the flow.

**Figure 2:**
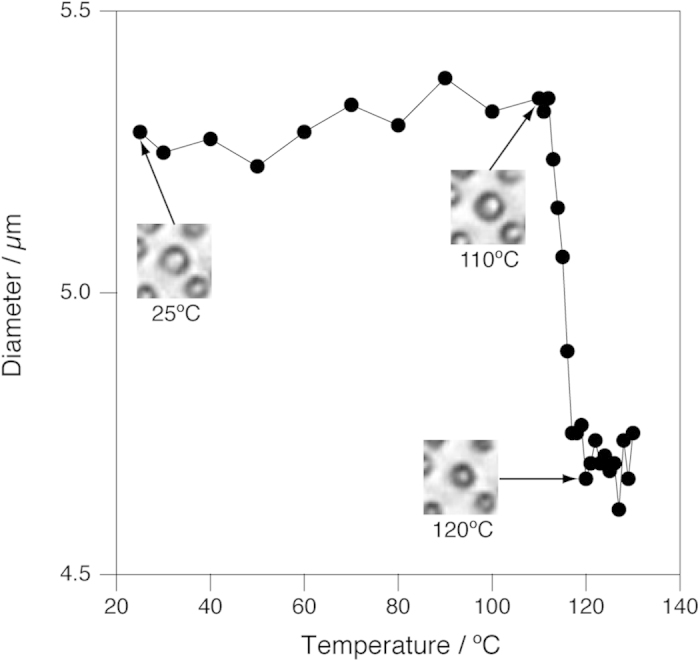
Change of the size of a *C. liquefaciens* cell as a function of temperature. Insets show microscopic images corresponding to the temperature of the data points indicated by arrows. Each images are 26 μm × 26 μm. A video clip showing the whole process is available in Movie S2.

When *C. liquefaciens* cells were heated in water from room temperature, spherical cells shrank slightly at around 120 °C. A typical example is shown in Fig. 2, in which the diameter of a cell, which was obtained by analysing images by image analysis software, is plotted against temperature together with selected microscopic images. In this particular case, the diameter of the cell abruptly decreased from 5.3 μm to 4.7 μm at temperatures between 112 °C and 117 °C. The shrinkage was observed for all the cells examined (see Movie S2). For the majority of the cells, the shrinkage occurred within a narrow temperature range between 115 and 120 °C (Figure S2).

**Figure 3:**
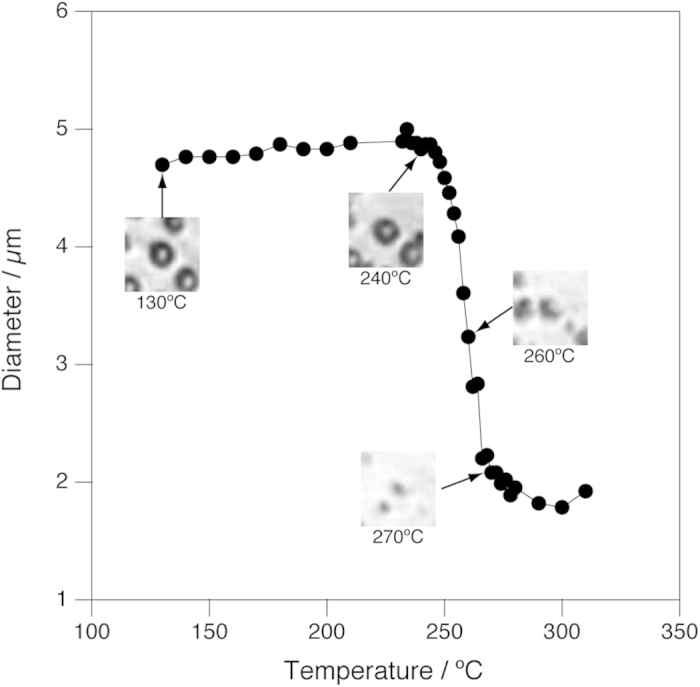
Change of the size of a *C. liquefaciens* cell in water between 130 °C and 310 °C as a function of temperature. Pressure was kept constant at 25 MPa. Insets show microscopic images corresponding to the temperature of the data points indicated by arrows. Each images are 26 μm × 26 μm.

Further heating revealed the spherical structure of the *C. liquefaciens* cells was retained up to 250 °C. The cells, which were adhered to the optical window at low temperatures, came off from the window at around 200 °C, and were swept away by the convective flow (Movie S2). The cells re-adhered onto the diamond surface at around 230 °C and stopped being flown. At temperatures between 250 °C and 270 °C, all the spherical cells shrank abruptly (Figure S3, see also Movie S2). Figure 3 shows the change of the diameter of a *C. liquefaciens* cell between 130 and 310 °C together with corresponding microscopic images. It is clearly seen that the diameter remained essentially unchanged between 130 and 250 °C, but decreased abruptly by more than 50% between 250 and 270 °C. We were not able to determine the final fate of the residue at higher temperatures because of the optical resolution of the microscopic system.

### Morphological change of cells of winter mushrooms in hydrothermal conditions

*In situ* high-resolution optical microscopy was also applied to examine another fungal cell, hyphae of *Flammulina velutipes* (winter mushrooms). *F. velutipes* was frozen in liquid nitrogen and pulverized with an aid of a mortar and a pestle. The frozen powder was dispersed in water and introduced into the sample chamber. Observation was made while heating the specimen from room temperature up to 400 °C under a constant pressure of 25 MPa (Video showing entire process is available in Movie S3). In this experiment, the temperature increased linearly with time between 100 °C and 320 °C at a rate of 35 °C/min, but the heating rate slowed down above 320 °C (Figure S4).

**Figure 4:**
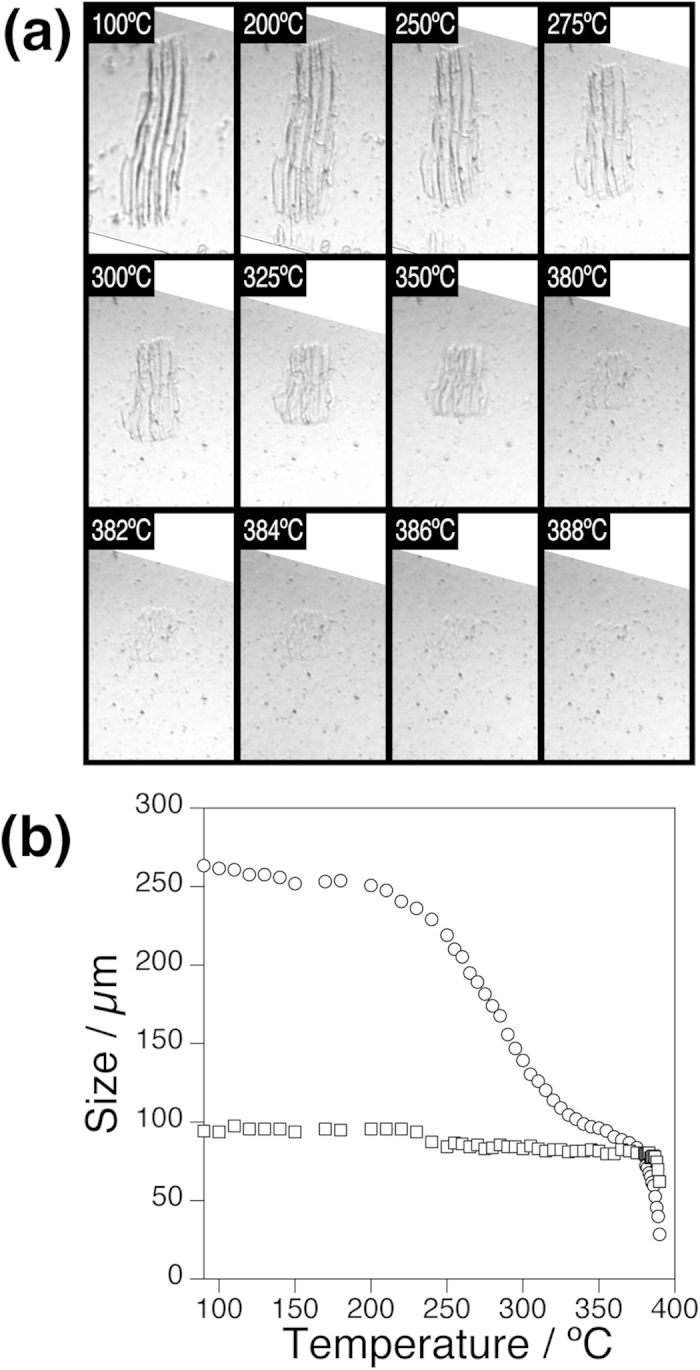
Morphological change of *F. velutipes* cells in hydrothermal conditions. **a**) A series of *in situ* high-resolution optical microscopic images showing hyphae of *F. velutipes* between 100 °C and 388 °C and at a constant pressure of 25 MPa. Each images are 327 μm × 192 μm. A video clip showing the whole process is available in Movie S3. (**b**) Change of length (circle) and width (square) of hyphae of *F. velutipes* as a function of temperature.

The large size of the hyphae allowed us to follow the cell morphology in more detail at higher temperatures (Fig. 4a). The hyphae remained essentially unchanged up to approximately 200 °C. Upon further heating, the hyphae underwent a highly anisotropic morphological change with temperature. It shrank dramatically along the long axis, whereas the width of the hyphae remained unchanged. The hyphae eventually disappeared completely between 380 °C and 390 °C.

To analyse the observation quantitatively, the length and width of the hyphae was measured by image-analysis software (Fig. 4b). The temperature dependent morphological change of the *F. velutipes* hyphae can be divided into three regimes. At temperatures below 250 °C, the length of the hyphae showed slight and monotonic decrease on temperature. It then decreased steeply between 250 °C and 380 °C. An inflection point was observed at around 320 °C, but we believe this is an artefact due to the slowing down of the heating rate (Figure S4). The width of the hyphae, on the other hand, remained essentially unchanged in these temperature regimes. Finally, above 380 °C, the residue dissolved rapidly in water and disappeared completely at 390 °C. The anisotropic shrinkage may be related to deformation of highly porous honeycomb-like structures that were observed by SEM for the interior tissue of *F. velutipes* stipes^27^.

## Discussion

It was reported that chitin was 3 times less reactive than cellulose when both polysaccharides were subjected to hydrothermal decomposition^28^. Our *in situ* microscopic observations also show that chitin is more resistant than cellulose in hydrothermal conditions and qualitatively support the previous observation.

Recalcitrance of chitin, as well as cellulose, in hydrothermal conditions does not mean that β-1-4 glycosidic linkage that connect *N*-acetylglucosamine or glucose units to make up the polysaccharide chain are resistant to hydrolysis in water at such high temperatures. A typical synthetic polymer, polystyrene, underwent pyrolytic decomposition in water above 360 °C, suggesting that even C—C covalent bonds do not remain intact^29^.

In the case of cellulose, robust crystalline structure remains intact in water at temperatures above 250 °C and effectively inhibit hydrolytic attacks of surrounding water molecules to the polysaccharide chains^21^. Once crystalline cellulose is transformed to an amorphous state and extensive hydrogen bonding networks are disrupted, the cellulose chains become accessible to high-temperature water and are hydrolysed rapidly.

It seems that dissolution of chitin in supercritical water proceeded in the same manner. Slow dissolution of the thin flake of chitin in water at 390 °C was associated with large deformation (roll-up of the flat flake of chitin, Movie S2). Similar deformation was observed for cellulose when it was transformed from a crystalline state to an amorphous state in water at high temperatures and high pressures^20^. Upon transformation, rigidity of crystalline cellulose was lost because extensive hydrogen-bonding networks that bound the cellulose chains tightly in crystals were disrupted. Thus, the deformation of chitin suggests that crystalline chitin also transformed to an amorphous state in water at around 380 °C. The higher transformation temperature of chitin may be ascribed to strong intramolecular hydrogen bonds that are formed between NH-COCH_3_ groups^28^.

Three crystalline isomorphs are known for chitin^30^. α-chitin is the most abundant isomorph, and occurs in fungal cell walls, in the crustacean exoskeletons, and in the insect cuticle^31^. β-chitin is found in squid pens^31^ and tube worms^32^. The chitin chains are packed alternately antiparallel in α-chitin, whereas they are all parallel in β-chitin^30^. The third form, γ-chitin, has been reported for cocoon fibers of the *Ptinus* beetle and the stomach of *Loligo*^30^. Unlike α- and β-chitin, two chitin chains run in one direction and another chain runs in the opposite direction in γ-chitin^30^.

The chitin chains are organized in sheets and held together via intra-sheet hydrogen bonds. There are also inter-sheet hydrogen bonds in α-chitin, whereas β-chitin lacks inter-sheet hydrogen bonds. Accordingly, β-chitin is more susceptible than α-chitin to intra-crystalline swelling and hydrolysis^31^. It is likely that the difference should also affect their stability in hydrothermal conditions.

In the case of cellulose, no significant difference was found when the stability of two polymorphs (cellulose-I and cellulose-II) in hydrothermal conditions was compared by *in situ* optical microscopy^21^. Presumably, the effect of the polymorphs on the hydrothermal stability of crystalline polysaccharides is not very significant and *in situ* optical microscopy is not sensitive enough to observe the effect.

The complete dissolution of the hyphae of *F. velutipes* (Fig. 4) and the dissolution of chitin (Fig. 1) were observed at almost the same temperature (380–390 °C). The results strongly suggest that chitin that makes up fungal cell wall is robust and remains intact up to ~380 °C. In fungal cell walls, chitin exists as fine bundles, 20–30 nm in width^33^. It is possible that chitin in a form of fine bundles may show different stability in hydrothermal conditions compared with a large crystal examined in this study due to the huge difference in the specific surface area. In the case of cellulose, however, large crystals (μm in size) and nanofibers (20–50 nm thick) showed similar stability in hydrothermal conditions because the crystalline structure of cellulose is essential in the stability in hydrothermal conditions and the surface effect does not affect the stability of cellulose^21^. Considering also the importance of crystalline structure in stability of chitin in supercritical water, it is likely that nanofibers of chitin may also show similar stability in supercritical water.

It is not clear from the present observations alone that other morphological changes including the slight shrinkage at around 120 °C (Fig. 2) and total collapse at around 250 °C (Fig. 3) of the *C. liquefaciens* cells and the anisotropic shrinkage of the hyphae of *F. velutipes* between 250 °C and 380 °C (Fig. 4). However, it is worth pointing out that the anisotropic shrinkage of the hyphae of *F. velutipes* started at almost the same temperature (~250 °C) as the spherical cells of *C. liquefaciens* collapsed, suggesting that these two changes may be triggered by the same mechanism.

Natural chitin occurs associated to other structural polymers such as proteins^34^. Hydrolysis of protein in hydrothermal conditions was studied by using bovine serum albumin (BSA) as a model^35^. Hydrolysis of BSA under hydrothermal conditions was rapid, and the highest amino acid yield at 270 °C was obtained after reaction for 90 s. Degradation of amino acids also occurred. Thus, hydrolysis of proteins in the cell walls could be a possible mechanism that triggered the anisotropic shrinkage of the *F. velutipes* hyphae and collapse of the *C. liquefaciens* cells.

Our observation may have implications in considering microbial diversity in high-temperature habitats such as deep-sea hydrothermal vents^36^. A hyperthermophilic archaeon, *Methanopyrus kandleri* strain 116, proliferates even at 122 °C, which is the known upper temperature limit of life^36^. Thermophily in known fungi is usually not as extreme as prokaryotes^37^, but presence of hyperthermophilic eukaryotes, which may survive brief exposure to temperatures above 190 °C, is inferred in hydrothermal sediment^38^. Inactivation of microorganisms after brief exposure to lethally high temperature occurs via rupture of the cell envelope^39^, and it is likely that the chitinous cell envelope of fungal cells is robust enough to withstand brief exposure to 190 °C.

In summary, *in situ* high-resolution optical microscopy revealed remarkable recalcitrance of chitin in hydrothermal conditions. The recalcitrance appears to play a key role in structural robustness of fungal cell morphology in hydrothermal conditions. Cellular morphology of two fungi, *F. velutipes* and *C. liquefaciens*, was retained at temperatures above 200 °C. Robustness seems to stem from the recalcitrance of the major component of fungal cell walls, chitin.

The findings have direct ramifications in considering fossilization of chitin-containing soft-bodied organisms and also in developing hydrothermal synthesis of chitin-based inorganic-organic composites. To that end, the microscopic observations should be complemented by other techniques such as X-ray diffraction to elucidate detailed molecular mechanisms behind the stability of chitin in hydrothermal conditions.

## Methods

### Materials

Chitin from club shell was obtained from Nacalai Tesque, Inc. (Kyoto, Japan) and used as received. A deep-sea yeast, *Cryptococcus liquefaciens* strain N6^40^, was kindly supplied by Prof. Fumiyoshi Abe, Aoyama Gakuin University. Cultivated *Flammulina velutipes* was purchased from a local glossary store.

### Microscopic Observation

Observations were made on an optical microscope equipped with a high-temperature and high-pressure sample chamber^23^,^26^. The instrument allows *in situ* observation of a specimen at temperatures up to 400 °C and pressures up to 35–40 MPa with an optical resolution of 2 μm. Detailed description of the instrument can be found elsewhere^23^.

Flakes of chitin were dispersed in water and introduced into the sample chamber. *C. liquefaciens* strain N6 was preincubated in yeast extract/peptone/dextrose (YPD) medium at 25 °C overnight. The cells were harvested by centrifugation, redispersed in water, and introduced into the sample chamber. The cells in the chamber were set overnight at room temperature, during which the cells adhered onto the lower optical window made of diamond. Excess cells were washed out by flowing water, and observation was made for the cells that adhered to the window. *F. velutipes* was frozen in liquid nitrogen and pulverized with an aid of a mortar and a pestle. The frozen powder was dispersed in water and introduced into the sample chamber for examination.

After loading the specimen, the entire system was pressurized to 25 MPa by introducing water by using an HPLC pump (PU-1580, JASCO, Hachioji, Japan). The pressure was controlled by a back-pressure regulator (Model 880-81, JASCO). The specimen was heated while maintaining the pressure constant at 25 MPa. Imaging was done by using a colour chilled CCD camera (C5810, Hamamatsu Photonics K. K., Hamamatsu, Japan) onto a VCR (GV-D300, SONY, Tokyo, Japan).

Due to the flow type design of the sample chamber, it was not possible to recover the specimen after observation, and end products of the specimen after observation were not analysed in this study.

### Image Analysis

When the observation was completed, the recorded video was transferred to a computer digitally via IEEE1394a. All of video editing including superimposition of a scale bar was done by Final Cut Pro (Apple Inc., Cupertino, CA). The length calibration of the video images was done by measuring the size of monodisperse polystyrene latices (mean diameter, 31 μm, Seradyne, USA). The image analysis was done by using ImageJ^41^.

## Additional Information

**How to cite this article**: Deguchi, S. *et al.* *In situ* microscopic observation of chitin and fungal cells with chitinous cell walls in hydrothermal conditions. *Sci. Rep.* **5**, 11907; doi: 10.1038/srep11907 (2015).

## Supplementary Material

Supplementary Information

Supplementary Movie S1

Supplementary Movie S2

Supplementary Movie S3
