# Supplementary material for: In situ microscopic observation of chitin and fungal cells with chitinous cell walls in hydrothermal conditions
Source: Sci Rep. 2015 Jul 7;5:11907. doi: 10.1038/srep11907 (PMC4493705; doi:10.1038/srep11907)
Supplement: Supplementary Information [file srep11907-s1.pdf]

Supplementary Information for "*In situ* microscopic observation of chitin and fungal cells with chitinous cell walls in hydrothermal conditions" by Deguchi, S, Tsujii, K. & Horikoshi, K.

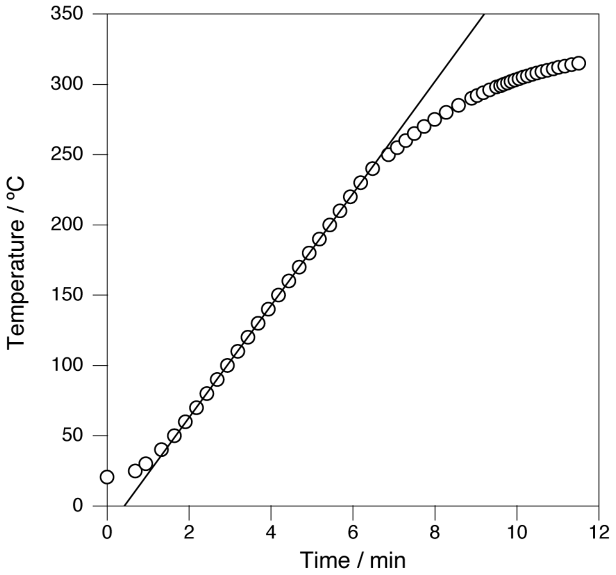

Figure S1. Temperature profile during observation of *C. liquefaciens* cells at temperatures up to 300ºC and at a constant pressure of 25 MPa.

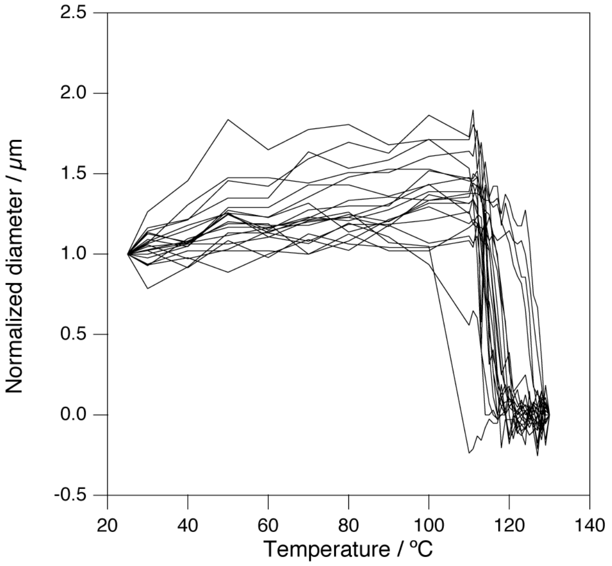

Figure S2. Change of the size of *C. liquefaciens* cells as a function of temperature. The figure compiles measurements of 20 different cells. For the majority of the cells, abrupt shrinkage was observed between 115 and 120 ºC. To make comparison easier, the measured values were normalized so that the value at the lowest measuring temperature (25ºC) was set to unity and that at the highest measuring temperature (130ºC) was set to zero.

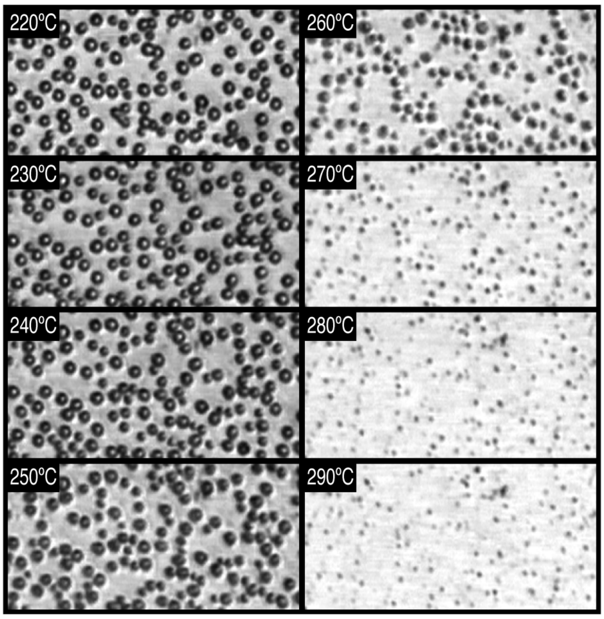

Figure S3. A series of *in situ* optical microscopic images of *C. liquefaciens* cells in water between 220ºC and 290ºC. Pressure was kept constant at 25 MPa. Each images are 197 µm × 99 µm. A video clip showing the whole process is available in Movie S2.

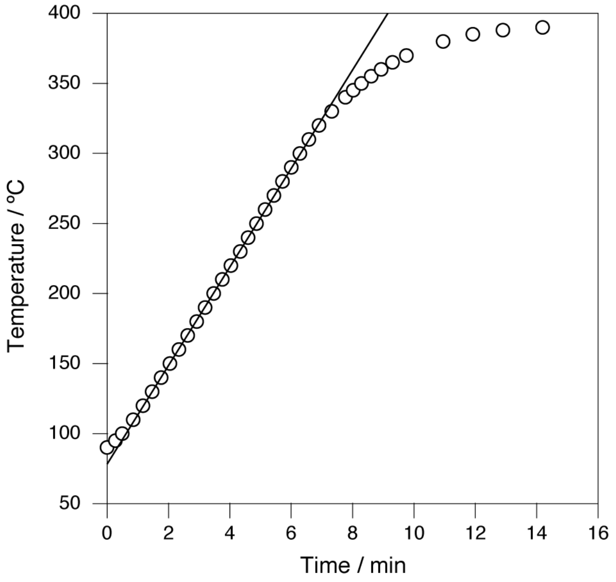

Figure S4. Temperature profile during observation of *F. velutipes* at temperatures up to 400ºC and at a constant pressure of 25 MPa.

Movie captions

Movie S1. A video clip showing behaviours of chitin in supercritical water. Pressure was kept constant at 25 MPa during the observation. The movie has been edited to 40 times the original playback rate.

Movie S2. A video clip showing behaviours of *C. liquefaciens* cells in water at high temperatures and at a constant pressure of 25 MPa during the observation. The movie has been edited to 40 times the original playback rate.

Movie S3. A video clip showing behaviours of *F. velutipes* in water at high temperatures and at a constant pressure of 25 MPa during the observation. The movie has been edited to 40 times the original playback rate.
